# Efficacy of PECS block in addition to multimodal analgesia for postoperative pain management in patients undergoing outpatient elective breast surgery: A retrospective study

**DOI:** 10.3389/fmed.2022.975080

**Published:** 2022-08-15

**Authors:** Alberto A. Uribe, Tristan E. Weaver, Marco Echeverria-Villalobos, Luis Periel, Joshua Pasek, Juan Fiorda-Diaz, Marilly Palettas, Roman J. Skoracki, Stephen J. Poteet, Jarrett A. Heard

**Affiliations:** ^1^Department of Anesthesiology, The Ohio State University Medical Center, Columbus, OH, United States; ^2^Department of Biomedical Informatics, The Ohio State University, Center of Biostatistics, Columbus, OH, United States; ^3^Department of Plastic Surgery, The Ohio State University Medical Center, Columbus, OH, United States

**Keywords:** nerve block, breast surgery, analgesics, opioid, PECS, regional anesthesia

## Abstract

**Background:**

Pectoralis nerve blocks (PECS) have been shown in numerous studies to be a safe and effective method to treat postoperative pain and reduce postoperative opioid consumption after breast surgery. However, there are few publications evaluating the PECS block effectiveness in conjunction with multimodal analgesia (MMA) in outpatient breast surgery. This retrospective study aims to evaluate the efficacy of PECS's blocks on perioperative pain management and opioid consumption.

**Methods:**

We conducted a retrospective study to assess the efficacy of preoperative PECS block in addition to preoperative MMA (oral acetaminophen and/or gabapentin) in reducing opioid consumption in adult female subjects undergoing outpatient elective breast surgery between 2015 and 2020. A total of 228 subjects were included in the study and divided in two groups: PECS block group (received PECS block + MMA) and control Group (received only MMA). The primary outcome was to compare postoperative opioid consumption between both groups. The secondary outcome was intergroup comparisons of the following: postoperative nausea and vomiting (PONV), incidence of rescue antiemetic medication, PACU non-opioid analgesic medication required, length of PACU stay and the incidence of 30-day postoperative complications between both groups.

**Results:**

Two hundred and twenty-eight subjects (*n* = 228) were included in the study. A total of 174 subjects were allocated in the control group and 54 subjects were allocated in the PECS block group. Breast reduction and mastectomy/lumpectomy surgeries were the most commonly performed procedures (48% and 28%, respectively). The total amount of perioperative (intraoperative and PACU) MME was 27 [19, 38] in the control group and 28.5 [22, 38] in the PECS groups (*p* = 0.21). PACU opioid consumption was 14.3 [7, 24.5] MME for the control group and 17 [8, 23] MME (p = 0.732) for the PECS group. Lastly, the mean overall incidence of postsurgical complications at 30 days was 3% (*N* = 5), being wound infection, the only complication observed in the PECS groups (*N* = 2), and hematoma (*N* = 2) and wound dehiscence (*N* = 1) in the control group.

**Conclusion:**

PECS block combined with MMA may not reduce intraoperative and/or PACU opioid consumption in patients undergoing outpatient elective breast surgery.

## Introduction

Breast surgery is one of the most common type of surgery worldwide (1). Around 30–60% of patient undergoing breast surgery reports moderate to severe acute pain and up to 43% of them experience persistent postoperative pain lasting 2–18 weeks, regardless of the surgical technique and/or the use of multimodal analgesia (MMA) (1–12). Effective management of acute postoperative pain has a significant impact on patient's immediate and long-term recovery and/or quality of life (2, 3, 13). A poorly controlled perioperative pain management strategy on this surgical population, may result in delayed functional recovery, delayed post anesthesia care unit (PACU) discharge and/or extended length of hospital stay with subsequent financial burden (1). In addition, inadequate postoperative pain management is recognized as one of the most relevant risk factors for the development of chronic postoperative breast pain (2, 3, 13).

Despite the implementation of novel surgical techniques and MMA regimens, postoperative pain remains one of the main perioperative concerns in patients undergoing breast surgeries (12). Consequently, regional blocks (thoracic epidural and paravertebral blocks) for breast surgery have been implemented as “gold standard” analgesic techniques in the perioperative settings despite their association to several adverse events (1, 12, 14, 15). These regional blocks have been associated with reduced surgical stress response, perioperative opioid consumption, and postoperative pain scores, which have had a significant impact on other perioperative outcomes, such as postoperative nausea and vomiting (PONV), pulmonary complications and PACU length of stay (1, 14, 15). Numerous studies have been published describing the effectiveness of Pectoralis nerves (PECS) blocks on postoperative pain and postoperative opioid consumption after cancer breast surgery (12, 16–28). However, there are few published reports evaluating the PECS block's effectiveness in non-cancer related breast surgery (28–31). PECS block I was first described in 2011 by *Blanco et al*. as an interfascial regional block for breast surgery that administers local anesthetic at the level of the third rib on the anterior chest wall between the pectoralis major and pectoralis minor muscles, targeting the medial and lateral pectoral nerves (15, 32). PECS II block involves the injection technique used in PECS I and a second injection of local anesthetic over the fourth rib on the anterior chest wall in the fascial plane between the serratus anterior muscle and pectoralis minor muscles, targeting the lateral branches of the T2–6 intercostal nerves; this variation allows PECS II to have an extended dermatome coverage anesthetizing the whole breast and axilla (15, 21, 28, 33, 34). We summarized the characteristics of PECS I and PECS II in Table 1.

**Table 1:** Characteristics of PECS I and II blocks.

**PECS Type**	**Nerves blocked**	**Muscular fascial planes involved**	**Indications**
PECS I	Lateral pectoral nerve Medial pectoral nerve	Pectoralis major muscle Pectoralis minor muscle	Subpectoral prosthesis/breast expanders/implant insertion
			Subpectoral ICD or pacemaker insertion
			Adjunct to paravertebral block following mastectomy
PECS II	Lateral pectoral nerve Medial pectoral nerve Lateral and anterior branch of T2–T6 spinal nerves Antero-cutaneous branches of intercostal nerves 3–6 Long thoracic nerve (C5–C7) Thoracodorsal nerve (C6–C8)	Pectoralis major and minor muscles Serratus anterio Axillary region: Teres major, Subscapularis, Latissimus dorsi	Mastectomy with or without reconstruction/subpectoral implant insertion Wide local excision of breast. Sentinel node biopsy. Axillary clearance. Submuscular breast prosthesis Pacemakers and implantable cardiac defibrillators Shoulder surgeries (involving armpit) Arteriovenous fistula formation high up in the arm/armpit

*T, thoracic; C, cervical; ICD, internal cardioverter defibrillators*.

Furthermore, for the last two decades there has been an increasing emphasis on promoting the use of MMA, particularly in the context of postoperative enhance recovery after surgery (ERAS) protocols, reducing perioperative opioid consumption and, subsequently, their side effects (35–37). The use of oral gabapentinoids and acetaminophen alone or in conjunction with regional anesthesia as part of MMA, has shown an adequate reduction on pain scores and opioid consumption (38, 39). Controversially, recent literature suggests that the reduction of opioid consumption associated to the use of perioperative gabapentinoids is not often clinically relevant (40).

Therefore, our study hypothesized that the use of a PECS block in combination with MMA will reduce perioperative opioid consumption in patients undergoing outpatient elective breast surgery. Considering the limited evidence on the use of PECS block in combination with MMA on breast surgery, we conducted a retrospective chart review to compare postoperative opioid consumption (oral morphine milligram equivalents [MME]) in subjects undergoing outpatient elective breast surgery under general anesthesia, preoperative oral MMA (with acetaminophen and/or gabapentin) and with or without PECS block.

## Methods

After full-board protocol review and approval (Protocol #2019E0641) from our Institutional Review Board (IRB), Office of Responsible Research Practices (ORRP)—The Ohio State University, we conducted a retrospective, single-center, observational, electronic medical record (EMR) review to assess the efficacy of using preoperative PECS block in addition to preoperative MMA with oral acetaminophen and/or gabapentin to reduce perioperative opioid consumption in adult female subjects undergoing outpatient elective breast surgery under general anesthesia at The Ohio State Wexner Medical Center (OSUWMC) between July 1, 2015 and June 26, 2020.

The decision of performing the PECS block prior to surgery was at the surgeon's and anesthesia care provider's discretion.

### Study population

The study included 228 female subjects, ≥18 years of age who underwent outpatient elective breast surgery and received preoperative MMA with oral acetaminophen and/or gabapentin as preventive analgesia, with or without PECS block. Subjects were excluded if they met any of the following criteria: chronic use of opioids due to any medical/surgical conditions, opioid consumption within 48 h prior to surgery, use of gabapentin within 30 days prior to surgery, use of acetaminophen within 7 days prior to surgery, pregnant women, subjects under legal protection, prisoners, and subjects scheduled for non-elective breast surgery. Eligible subjects were allocated into one of two groups: PECS block Group (both, PECS and MMA were administered) and control group (only MMA was administered).

### Clinical outcomes

The primary outcome was to compare MME in subjects who underwent outpatient elective breast surgery and received oral MMA with or without the use of preoperative PECS block. Secondary outcomes included the length of surgery, length of anesthesia, and length of PACU stay, incidence of PONV, rescue antiemetic medication requirements, amount of non-opioid analgesic medication required during surgery and PACU, and incidence of 30-day postoperative complications.

### Anesthesia/analgesia technique

Preoperative MMA with oral acetaminophen 975 mg and/or gabapentin 600 mg as preventive analgesia was given within 2 h prior to surgery. The anesthesia technique followed institutional recommended guidelines. Induction was conducted with intravenous (IV) fentanyl 1.5–2.5 μg/kg and lidocaine 40–100 mg, followed by IV propofol 2.0–2.5 milligrams per kilo (mg/kg) as a hypnotic agent and IV rocuronium 0.6–1.0 mg/kg for the neuromuscular blockade to facilitate endotracheal intubation. Anesthesia maintenance was achieved with sevoflurane in a 45/55% oxygen/air mixture to attain and average minimum alveolar concentration (MAC) of 1 throughout the intraoperative period. Intraoperative opioids included intravenous fentanyl and hydromorphone, while oral oxycodone was prescribed after PACU/hospital discharge.

### PECS block technique

The ultrasound-guided PECS block was performed following institutional recommended guidelines, immediately after anesthesia induction. A local anesthetic infiltration was performed at the levels of 3rd and 4th ribs, along the mid-axillary line from each side. PECS I was performed by introducing the needle in plane from medial to lateral and injecting 20–30 ml of 0.5% ropivacaine in the interfascial plane between pectoralis minor and pectoralis major muscles form each side. PECS II blocks consisted of the same steps as PECS I block, but with the bilateral infiltration of the local anesthetic between the pectoralis minor and serratus anterior muscles.

### Statistical analysis

Patient demographic and clinical characteristics were summarized for the two study groups using descriptive statistics. Comparisons between the control and PECS block groups included baseline demographics, pre-operative/intra-operative medications, surgery types, postoperative opioid consumption, and patient outcomes. Categorical variables were compared between groups using either a Chi-square test or a Fisher's Exact test, and continuous variables were compared using either a two-sample *t*-test or a Wilcoxon Rank Sum test. Linear regression analysis was also used to assess the association of overall opioid consumption between both groups, adjusted by length of surgery and type of procedure. Secondary objectives (time to first opioid dose, incidence of PONV, total PACU stay length) were compared between the two groups using either a Chi-square test, Fisher's Exact test, two-sample *t*-test, or a Wilcoxon Rank Sum test, where appropriate. All data analyses were performed using SAS 9.4 (SAS Institute Inc., Cary, NC) or Stata 14 (StataCorp LLC, College Station, TX).

## Results

### Study participants and clinical characteristics

**Figure 1:**
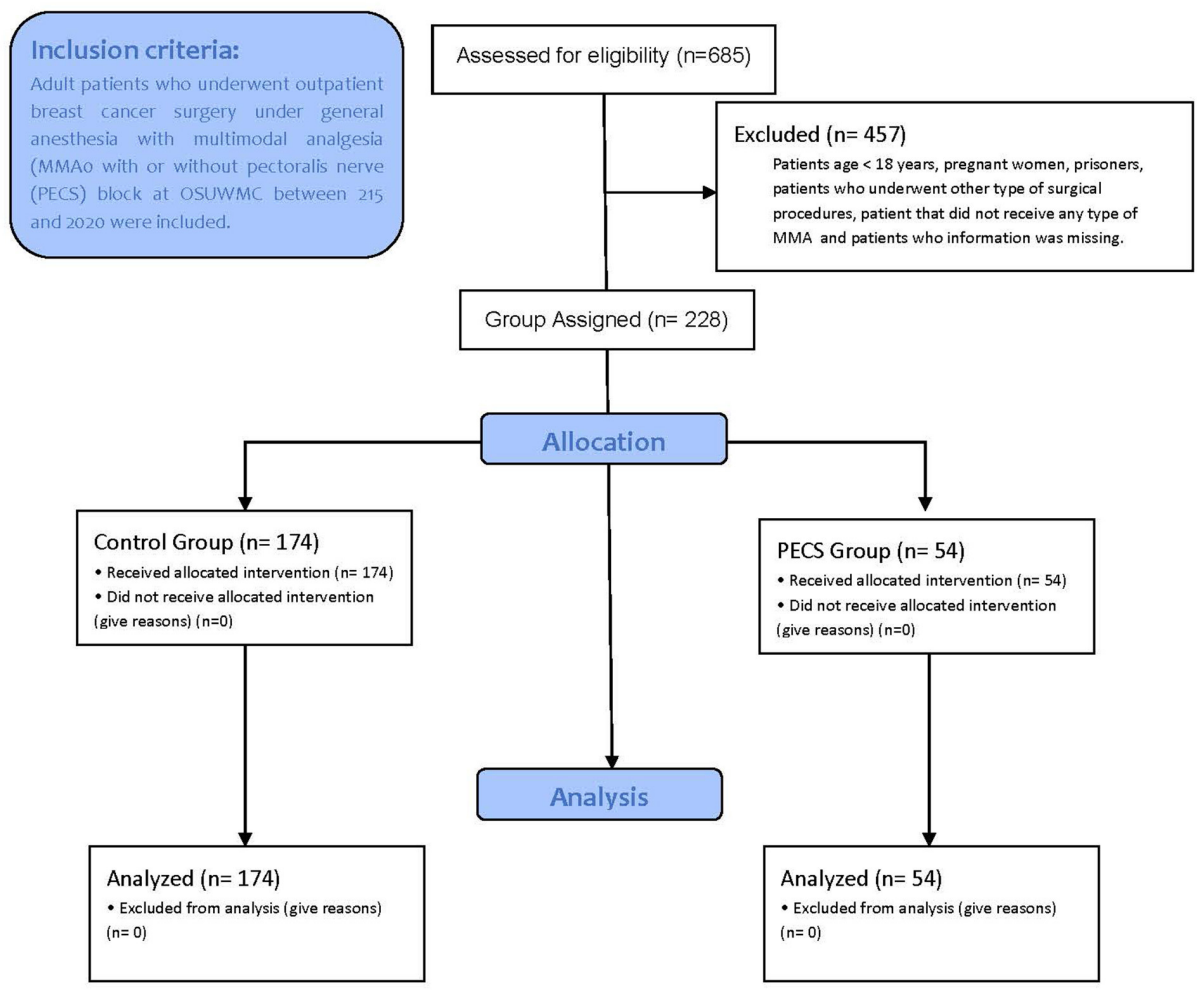
CONSORT flow diagram for subjects included in the study. N, number of participants; MMA, multimodal analgesia.

A total of 685 subjects that underwent outpatient elective breast surgery under general anesthesia at The Ohio State Wexner Medical Center (OSUWMC) between July 1, 2015 and June 26, 2020 were screened to confirm eligibility criteria. Consequently, a total of 457 subjects were excluded due age <18 years, pregnant women, prisoners, subjects who underwent other type of surgical procedures, patient that did not receive any type of MMA and subjects who relevant information was missing. Therefore, a total of 228 eligible subjects were included in the study for statistical analysis. Fifty-four (*n* = 54) subjects were allocated into the PECS group and 174 subjects were allocated in the control group (Figure 1).

Demographics and surgical variables are summarized in Table 2. The average age of subjects was 48 ± 15.1 years old, with subjects in the control group slightly older in age than in the PECS group [50.4 ± 14.2 versus (vs.) 40.3 ± 15.7; *p* < 0.001]. The groups were comparable with respect to body mass index (BMI) (30 [6.2]). The PECS group had fewer American Society of Anesthesiology physical status (ASA) classification of 3 (9.3 vs. 27.6%; *p* = 0.001) and more ASA 1 classification of 1 (33.3 vs. 14.3%; *p* = 0.001) when compared with the control group. ASA classification of two subjects was similar in both groups.

**Table 2:** Demographics and clinical variables.

**Variables**	**Control (*N* = 174)**	**PECS Block (*N* = 54)**	**Total (*N* = 228)**	***P*-value**
Age, years, mean (SD)	50.4 (14.2)	40.3 (15.7)	48 (15.1)	**<0.001**
Weight, kg, mean (SD)	80.2 (17.5)	80.2 (15)	80.2 (16.9)	0.996
Height, meters, mean (SD)	1.6 (0.1)	1.6 (0.1)	1.6 (0.1)	0.219
BMI, kg/m^2^, mean (SD)	29.9 (6.5)	30.3 (5.4)	30 (6.2)	0.652
**ASA physical status**, ***N*** **(%)**				**0.001**
I	25 (14.3%)	18 (33.3%)	43 (18.9%)	
II	101 (58%)	31 (57.4%)	123 (53.9%)	
III	48 (28%)	5 (9%)	53 (23%)	
History of PONV or motion sickness, *N* (%)	42 (24%)	9 (17%)	51 (22%)	0.25
MMA pre-op with gabapentin alone, *N* (%)	3 (1.7%)	1 (1.9%)	4 (1.8%)	0.132
MMA pre-op with acetaminophen alone, *N* (%)	31 (17.8%)	5 (9.3%)	36 (15.8%)	0.95
MMA pre-op gabapentin + acetaminophen, *N* (%)	140 (80%)	48 (89%)	188 (82%)	0.155
MMA pre-op gabapentin dose, mg, median (IQR)	600 [300, 900]	600 [300, 900]	600 [300, 900]	**0.132**
MMA pre-op acetaminophen dose, mg, median (IQR)	650 [650, 975]	975 [650, 975]	975 [650, 975]	**0.95**
**Intraoperative intravenous medication**				
Dexamethasone, *N* (%)	153 (88%)	53 (98%)	206 (90%)	**0.032**
Dexamethasone, mg, median (IQR)	8 (4, 8)	8 (4, 8)	8 (4, 8)	0.859
Ondansetron, *N* (%)	166 (95%)	53 (98%)	219 (96%)	0.69
Ondansetron, mg, median (IQR)	4 (4, 4)	4 (4, 4)	4 (4, 4)	0.599
Ketamine, *N* (%)	18 (10%)	7 (13%)	25 (11%)	0.56
Ketamine, mg, median (IQR)	30 [30, 50]	50 [30, 50]	40 [30, 50]	0.824
Fentanyl, *N* (%)	170 (98%)	53 (98%)	223 (98%)	0.341
Fentanyl, ucg, median (IQR)	125 [100, 200]	150 [100, 200]	125 [100, 200]	0.341
Hydromorphone, *N* (%)	66 (38%)	27 (50%)	93 (41%)	0.115
Hydromorphone, ucg, median (IQR)	1 [1, 2]	1 [1, 2]	1 [1, 2]	0.202
Ketorolac, *N* (%)	14 (8%)	10 (19%)	24 (11%)	**0.029**
Ketorolac, mg, median (IQR)	30 [30, 30]	30 [30, 30]	30 [30, 30]	0.074
**Pectoral nerves (PECS) block type**, ***N*** **(%)**				
PECS I bilateral		5 (9.3%)		
PECS II bilateral		38 (70.4%)		
PECS II unilateral		5 (9.3%)		
PECS (unknown type)—bilateral		6 (11.1%)		
**Type of surgery**				
Breast reduction, *N* (%)	67 (39%)	42 (78%)	109 (48%)	**<0.001**
Mastectomy/lumpectomy, *N* (%)	60 (34%)	3 (6%)	63 (28%)	
Mastopexy, *N* (%)	14 (8%)	5 (9%)	19 (8%)	
Breast augmentation, *N* (%)	16 (9%)	2 (4%)	18 (8%)	
Breast reconstruction, *N* (%)	17 (10%)	2 (4%)	19 (8%)	
Length of surgery, min, mean (SD)	125 [77, 168]	153 [128, 182]	132 [93, 174.5]	**0.001**
Length of anesthesia, min, mean (SD)	163 [110, 211]	190 [164, 231]	175 [127, 221.5]	**0.004**
Length of PACU stay, min, mean (SD)	146 [125, 186]	141 [122, 168]	144 [124, 184.5]	0.501

*N, number; SD, standard deviation; PECS, pectoralis nerve block; BMI, body index mass; ASA, American Society of Anesthesiology physical status classification; PONV, postoperative nausea and vomiting; mg, milligram; IQR, interquartile range; ucg, microgram; PACU, Post-Anesthesia Care Unit; min, minutes; ucg, microgram; kg, kilogram; kg/m2, Kilogram-Meter Squared. Bold values indicate statistically significant results*.

Regarding the preoperative MMA administration, 140 (80%) subjects in the control group and 49 subjects (89%) in the PECS group received a combination of acetaminophen and gabapentin (*p* = 0.155); the remaining subjects in each group received either acetaminophen or gabapentin alone. The median dose of acetaminophen and gabapentin for all subjects was 975 [650–975] mg. and 600 [300–900] mg, respectively.

In the PECS group, the PECS II technique was the most used among subjects compared to PECS I (79.6 vs. 9.3%, respectively, *p* < 0.001). Moreover, a bilateral PECS block was performed in most subjects with only 5% of the PECS II group subjects having a unilateral PECS block.

Among the PECS group, the most common surgeries performed were breast reduction (78%), mastopexy (9%), and breast augmentation (4%). On the other hand, the most common surgeries performed on the control group were breast reduction (39%), mastectomy/lumpectomy (34%) and breast reconstruction (10%).

The median length of surgery time was significantly prolonged in the PECS group in comparison with the control group (153 [128–182] min and 125 [77–168] min, *p* < 0.001). Consequently, the median duration of anesthesia was also longer in the PECS group when compared with the control group (190 [53–293] min and 163 [34–717] min, *p* = 0.04). Lastly, the median length of PACU stay was similar between the control and PECS groups (146 [125–186] min vs. 141 [122–168], *p*: 0.501; respectively).

### Primary outcome

Intraoperative median MME was similar in both groups with a median value of 27 (19–38) mg in the control group and 28.5 (22–38) mg in the PECS group (*p* = 0.21). No significant differences between both groups were observed for PACU opioid consumption (14.3 [7–24.5] mg in the control group and 17 [8–23] mg in the PECS group; *p* = 0.732). Overall, there were not significant differences for opioid consumption during the entire perioperative period (i.e., intraoperative and PACU) between groups, (43.5 [31–61] mg in the control group and 45.5 [38–58.3] mg in the PECS group; *p* = 0.284) (Table 3).

**Table 3:** Perioperative opioid consumption.

**Variables**	**Control (*N* = 174)**	**PECS block (*N* = 54)**	**Total (*N* = 228)**	***P*-value**
Intraoperative opioid consumption, oral morphine mg, mean (SD)	27 [19, 38]	28.5 [22, 38]	27 [19, 38]	0.21
PACU opioid consumption, oral morphine mg, mean (SD)	14.3 [7, 24.5]	17 [8, 23]	15 [8, 23.8]	0.732
Overall opioid consumption, oral morphine mg, mean (SD)	43.5 [31, 61]	45.5 [38, 58.3]	45.5 [33, 60.5]	0.284

*N, number; PECS, pectoralis nerve block; SD, standard deviation; mg, milligram*.

Lastly, an additional linear regression analysis was conducted to assess the association of overall opioid consumption between both groups, adjusted by length of surgery and type of procedure. This analysis showed no differences between groups. Table 4 summarizes the adjusted OR, 95% CI, and associated *p*-values.

**Table 4:** Logistic regression analysis of overall opioid consumption adjusted by surgery length or surgery type.

**Variable**	**Level**	**Control (*n* = 131)**	**PECS block (*n* = 45)**	***P*-value**
Overall opioid consumption, MME	Mean (SD)	45.0 (2.9)	46.7 (4.0)	0.601

*N, number; SD, standard deviation; PECS, pectoralis nerve block; MME, oral morphine milligrams equivalents*.

### Secondary outcomes

There were a few differences on the use of intraoperative medications (Table 2). The use of intraoperative ketorolac (IV 30 mg) and dexamethasone (IV 8 mg) were significantly higher in the PECS group than in the control group (19 vs. 8%; *p* = 0.029 and 98 vs. 88%; *p* = 0.032, respectively).

The overall incidence of PONV in PACU was 18% and was slightly higher in the PECS group compared to the control group (17 and 22%, respectively; *p* = 0.41). PONV rescue medication was required in 19.3% of subjects who experienced PONV. Ondansetron was used in 14% of these subjects as a PONV rescue medication, whereas haloperidol was administered in the remaining 5% with no statistical differences between groups (Table 5). Lastly, the mean overall incidence of postsurgical complications at 30 days was very low (5 [3%]), with wound infection as the only complication observed in the PECS groups (*N* = 2), and hematoma (*N* = 2) and wound dehiscence (*N* = 1) in the control group (Table 5).

**Table 5:** Postoperative outcomes.

**Variables**	**Control (*N* = 174)**	**PECS block (*N* = 54)**	**Total (*N* = 228)**	***P*-value**
**PONV**				
PONV incidence, *N* (%)	30 (17%)	12 (22%)	42 (18%)	0.41
PONV rescue medication, *N* (%)	30 (17%)	12 (22%)	42 (18%)	0.41
**PONV rescue**, ***N*** **(%)**	32 (18.4%)	12 (22%)	44 (19.3%)	0.41
Rescue with Promethazine, *N* (%)	1 (1%)	0 (0%)	1 (0%)	0.577
Rescue with Ondansetron, *N* (%)	28 (16%)	4 (7%)	32 (14%)	0.109
Rescue with Haloperidol, *N* (%)	3 (2%)	8 (15%)	11 (5%)	**<0.001**
Ondansetron dose, mg, Median (IQR)	4 [4, 4]	4 [4, 4]	4 [4, 4]	0.712
Haloperidol dose, mg, median (IQR)	1 [1, 1]	1 [1, 1]	1 [1, 1]	0.999
**Postoperative complication (30 days)**				
Wound infection, *N* (%)	0	2 (100%)	2 (29%)	0.2
Wound dehiscence, *N* (%)	1 (20%)	0	1 (14%)	NA
Hematoma, *N* (%)	2 (40%)	0	2 (29%)	NA

*N, number; %, percentage; PECS, pectoralis nerve block; PONV, postoperative nausea and vomiting; mg, milligram. Bold values indicate statistically significant results*.

## Discussion

The results obtained in our study showed that the use of PECS as a strategy for postoperative analgesia after breast surgery did not decrease the perioperative opioid consumption when compared with the use of opioid-free MMA alone (acetaminophen/gabapentin/ketorolac). There were no significant differences in opioid consumption between groups during the intraoperative period, the PACU stay or the overall in-hospital perioperative period. In addition, when adjusted, overall opioid consumption by surgery length and type, there were no inter-group substantial differences either.

The results of our study differ slightly from some recently published evidence showing the efficacy of PECS block on reducing perioperative opioid use in subjects undergoing breast surgery. The vast majority of prospective and retrospective studies, systematic reviews, and meta-analysis published in recent years have shown that the combination of general anesthesia and PECS blocks reduces the severity of postoperative pain and perioperative opioid consumption, and positively impact other postoperative outcomes such as PONV and the length of hospital stay when compared to MMA strategies without loco-regional anesthesia techniques (16–19, 21–26, 30, 31, 41–45). Therefore, the addition of PECS blocks to general anesthesia may provide adequate postoperative analgesia and substantially reduce perioperative opioid consumption (17, 18, 24, 27, 30, 42, 43, 46).

A retrospective study by *Morioka et al*. in subjects who underwent breast cancer surgery under anesthesia with either total intravenous anesthesia (TIVA) + PECS or TIVA without PECS, showed a substantial reduction in the use of intraoperative remifentanil in the TIVA + PECS group compared with the group that received TIVA alone (TIVA: 10.9 ± 2.9 μg/kg/h; TIVA + PECS: 7.3 ± 3.3 μg/kg/h; *p* < 0.001) (27). However, the authors found no differences between groups in regard to the requirement of postoperative supplemental analgesia (TIVA: 24.3% [9/36]; TIVA + PECS: 17.1% [6/35]; *p* = 0.32) and the incidence of PONV (TIVA: 16.7% [6/36]; TIVA + PECS: 11.4% [4/35]; *p* = 0.39) (27). *Kim et al*. retrospectively studied the perioperative opioid consumption in 80 subjects who underwent breast conservative surgery plus sentinel lymph node biopsy. Forty subjects (*N* = 40) were allocated in the control group (balanced anesthesia) and 40 in the PECS II group (balanced anesthesia + PECS II) (42). The authors reported a reduced opioid consumption during the first 24 postoperative hours in the PECS II group when compared to the control group (43.8 ± 28.5 g vs. 77.0 ± 41.9 g; *p* < 0.001). However, the intergroup incidence of rescue analgesia was equivalent during the same period (42).

A recent single center, randomized control trial (RCT) compared the efficacy of PECS I block, local anesthetic wound infusion (LA infusion), or the combination of both for pain management after breast cancer surgery during a 24-h postoperative period (18). The results of the study showed that the combination of PECS + LA infusion was more effective than LA infusion alone or PECS alone to control postoperative pain (mean [(SD) 71 (34) vs. 58 (41) vs. 23 (20), respectively; *p* = 0.002]). Moreover, the PECS + LA combination was associated with a decreased opioid consumption in the first 24 h after surgery (18). Similarly, *Altiparmak et al*. studied the efficacy of PECS vs. erector spinae plane (ESP) block in terms of postoperative opioid (tramadol) consumption and pain levels measured by numerical rating scale (NRS). Postoperative consumption of tramadol was significantly lower in the PECS group (132.78 ± 22.44 mg in PECS group vs. 196 ± 27.03 mg in ESP group; *p* = 0.001) as well as NRS scores after 30 min and up to 24 h (43).

A recently published RCT by *Choi et al*. analyzed 39 subjects undergoing breast surgery under TIVA (propofol-remifentanil). Subjects were randomized to receive either TIVA + PECS II block with ropivacaine 0.5% (PECS group; *n* = 20) or TIVA alone (control group; *n* =18) (17). The authors concluded that not only the total remifentanil infused dose was much lower in the PECS group than in the control group (6.8 ± 2.2 μg/kg/h vs. 10.1± 3.7 μg/kg/h; *P* = 0.001), but also the rescue analgesic requirements in the PACU were lower in the PECS group (17). *Karaca et al*. recently studied the impact of PECS block in 54 subjects undergoing breast augmentation surgery. In this study, PECS block was performed in 27 subjects after general anesthesia induction (group P) while 27 subjects were the control group (group C) (30). Both groups received postoperative analgesia with patient-controlled analgesia (PCA)-fentanyl for up to 24 h after surgery. Fentanyl total doses, incidence of PONV, and PACU and hospital length of stay were analyzed. Authors reported that 24-h fentanyl consumption was significantly reduced in Group P when compared to Group C (378.7 ± 54.0 mg and 115.7 ± 98.1 mg, respectively: *p* < 0.001). Moreover, significant reductions were observed in pain levels (visual analog scale or VAS score), PONV incidence, and hospital LOS in Group P in comparison with Group C (30). A meta-analysis performed by *Zhao et al*. compared the effectiveness of general anesthesia + PECS II block (experimental group) vs. general anesthesia (GA) + sham block (control group) on intra- and postoperative opioid consumption (sufentanil, fentanyl, and remifentanil), incidence of PONV, postoperative pain scores up to 24-h, and requirements of opioids and non-opioids analgesic rescue medications (45). Compared to the GA group, the use of PECS block effectively reduced the intraoperative and postoperative use of opioids, the incidence of PONV, the need for postoperative rescue analgesia, and pain scores within 0–6 h after surgery. Nevertheless, a subgroup analysis showed no significant reduction on perioperative opioid consumption after a PECS II block (45). Lastly, a recent meta-analysis conducted by *Hussain et al*. evaluated the analgesic effectiveness of PECS II vs. control vs. paravertebral block in breast cancer surgery settings (28). The study analyzed the data from 14 RCT that included 887 subjects and concluded that PECS II reduced at least 30 mg of morphine consumption and in-rest pain during the first 24 h following breast cancer surgery when compared with the control group (28). In addition, there were not significant differences in all outcomes between the use of PECS and paravertebral block (28).

Conversely, some authors have reported similar results to our study, in which the use of PECS block did not significantly reduce perioperative opioid use when compared to MMA alone (29, 47, 48). A dual-centered, placebo-controlled RCT performed by *Cros et al*. in 128 subjects to evaluate the efficacy of ultrasound- guided PECS I vs. placebo in managing pain after unilateral cancer breast surgery, showed that there was no significant intergroup differences in intraoperative sufentanil consumption (20.0 [15.0–20.0] μg vs. 20.0 [15.0–25.0] μg, respectively; *p* = 0.8536) (47). Likewise, there were no statistical differences in PACU morphine consumption (1.5 [0.0–6.0] mg vs. 3.0 [0.0–6.0] mg; *p* = 0.20) and up to 24-h postoperatively (47). In a double-blinded, placebo-controlled prospective study conducted by *Lanier et al*. 47 subjects undergoing tissue expander/implant breast reconstruction were randomly allocated to either intraoperative PECS block with bupivacaine 0.25%, or a sham nerve block (control group) with normal saline (29). No statistical differences were reported between both groups in pain level, opioid consumption (8 vs. 17 MME; *p* = 0.26), quality of recovery, and antiemetic rescue medication during PACU stay and during hospitalization (92 vs. 114; *p* = 0.31) (29).

The overall incidence of PONV in our study population was 18%, with a slightly higher incidence in the PECS group than in the control group (22 vs. 17%, respectively; *p* = 0.410). No subjects experienced delayed hospital discharge, remained in the hospital after surgery due to PONV or were admitted after discharge due to delayed PONV (DPONV). The overall and between-group incidence of PONV in this study is lower than the prevalence reported in recent literature for ambulatory surgery (49, 50) and specifically for breast surgery (35, 45, 51, 52). The fact that opioid consumption in our study was comparable between groups most likely did not allow for a significant inter-group difference in PONV. Several clinical studies and meta-analysis showing a significant impact of PECS blocks on opioid consumption have reported a marked reduction in PONV frequency when compared with control groups or with MMA regimens without peripheral nerve block (19, 26, 28, 30, 31, 45, 48).

The prevalence rate of 30-day surgical complications was 3%, with no significant between-groups difference (3 vs. 4%). Hematoma (0.88%) and wound infection (0.88%) were the most common complications observed in that timeframe. In 2007, *El- Tamer et al.*, using the database of The National Surgical Quality Improvement Program Patient Safety in Surgery (NSQIP), reported that the most common 30-day complication after breast cancer surgery was wound infection (4.34%) (53). A later study by *Qin et al.*, also examining the data collected from NSQIP, reported the overall incidence of complications after breast cancer surgery was 5.4% (54). More recently, *Spataro et al.*, conducted a retrospective study from a secondary data repository which included a sample of 513,423 subjects and reported a 1.6% incidence of complications after ambulatory breast augmentation surgery (55).

Recent published evidence have questioned the benefits of using gabapentinoids in postoperative pain management regimens due to the high incidence of adverse effects such as sedation, dizziness, and visual disturbances that impede early mobilization and delay recovery; in addition, opioid-sparing effects of gabapentinoids have resulted clinically insignificant (40, 56–61). In addition, a few meta-analysis have been recently conducted to assess the effect of gabapentinoid in postoperative pain. *Chaparro et al*. conducted a Cochrane systematic review assessing trials that use perioperative gabapentin and ketamine in patients undergoing orthopedic and cardiac surgeries (59). The study suggested that the use of gabapentin did not significantly reduce postoperative pain when compared to placebo at 3 and 6 months and ketamine significantly reduced the incidence of chronic pain after surgery (59). Another meta-analysis conducted by *Clarke et al*. assessed the effect of perioperative use of gabapentinoids across different postoperative timepoints and concluded that its use could reduce the incidence of chronic pain (60). Lastly, another meta-analysis assessed acute and chronic pain in patients receiving preoperative pregabalin or gabapentin undergoing breast cancer surgery (61). The study concluded that gabapentin and pregabalin reduced opioid consumption in PACU, gabapentin reduces postoperative pain during the first 24 h after surgery and neither drug had an effect on reducing chronic postoperative pain (61).

We are aware of some limitations in our study that could increase the risk of bias in our results. First, due to the intrinsic limitation of a retrospective study, the small sample size, and the inability to collect opioid consumption for 24 h, limited our study to investigate an extended postoperative opioid consumption outcome that could provide us a better understanding of the analgesic needs and postoperative acute or chronic pain for this outpatient population. Second, most of study population received preoperative acetaminophen and gabapentin (82%) and intraoperative dexamethasone (90%) as part of the MMA regimen, and a few subjects (11%) received intraoperative ketorolac. Consequently, the doses of MMA regimen were not consistent among subjects because clinicians guided their clinical postoperative pain management according to institutional clinical guidelines, pre-existing medical conditions and/or their own or personalized clinical discretions could also play a role in this variability. Third, an important factor that could have influenced the slightly higher intraoperative opioid requirements in the PECs group is the longer duration of surgical procedures in the PECS block group when compared to the control group. Fourth, a potential human error during data collection and/or data transferring, as well as some inconsistencies among medical records may have occurred. Fifth, the recent implementation (2018) of PECS block use at our institution contributed to the uneven number of subjects analyzed on each group. Sixth, due to the retrospective research methodology of the study, we were not able to collect pain scores after surgery because the data was inconsistent on the number and time of pain scores assessed after surgery. Seventh, the inclusion of different breast procedures, mainly breast reduction and mastectomy/lumpectomy, with various degrees of invasiveness might reflect different trajectories of postoperative pain and opioid consumption might interfered the outcomes of this study. Finally, other factors that were not within the scope of our analysis, such as subjects' comorbidities, concomitant medication and/or pharmacodynamic considerations may had impact our outcomes.

## Conclusions

Despite the fact that intraoperative peripheral nerve blocks are commonly used as an adjunct safe approach for pain management, our results suggest that the use of PECS block combined with MMA may not reduce intraoperative and/or PACU opioid consumption in subjects undergoing outpatient elective breast surgery.

## Data availability statement

The data analyzed in this study is subject to the following licenses/restrictions: Available upon request. Requests to access these datasets should be directed to alberto.uribe@osumc.edu.

## Ethics statement

The studies involving human participants were reviewed and approved by Office of Responsible Research Practices (ORRP)—The Ohio State University. Written informed consent for participation was not required for this study in accordance with the national legislation and the institutional requirements.

## Author contributions

Conceptualization: AU, TW, MP, RS, SP, and JH. Data curation and investigation: AU, ME-V, LP, JP, JF-D, and JH. Formal analysis: AU, JF-D, and MP. Methodology and resources: AU and JH. Project administration: AU, ME-V, JF-D, and JH. Supervision: AU, TW, and JH. Validation: AU, TW, ME-V, LP, JP, and JH. Visualization: AU, ME-V, LP, JP, and JH. Writing original draft and writing review and editing: AU, TW, ME-V, LP, JP, JF-D, MP, RS, SP, and JH. All authors contributed to the article and approved the submitted version.

## Conflict of interest

The authors declare that the research was conducted in the absence of any commercial or financial relationships that could be construed as a potential conflict of interest.

## Publisher's note

All claims expressed in this article are solely those of the authors and do not necessarily represent those of their affiliated organizations, or those of the publisher, the editors and the reviewers. Any product that may be evaluated in this article, or claim that may be made by its manufacturer, is not guaranteed or endorsed by the publisher.

## Abbreviations

PECS: pectoral nerve block
MMA: multimodal analgesia
PACU: post-anesthesia care unit
MME: oral morphine milligram equivalents
EMR: electronical medical records
PONV: postoperative nausea and vomiting
MAC: minimum alveolar concentration
BMI: body mass index
ASA: American Society of Anesthesiologists physical status
LOS: length of stay
NSQIP: National Surgical Quality Improvement Program
IV: Intravenous.
